# Polyphenols for Livestock Feed: Sustainable Perspectives for Animal Husbandry?

**DOI:** 10.3390/molecules27227752

**Published:** 2022-11-10

**Authors:** Marialuisa Formato, Giovanna Cimmino, Nabila Brahmi-Chendouh, Simona Piccolella, Severina Pacifico

**Affiliations:** 1 Department of Environmental, Biological and Pharmaceutical Sciences and Technologies, University of Campania “Luigi Vanvitelli”, Via Vivaldi 43, 81100 Caserta, Italy; 2 Laboratory of 3BS, Faculty of Life and Nature Sciences, University of Bejaia, Bejaia 06000, Algeria

**Keywords:** cattle, polyphenols, large ruminants, fermentation parameters, biohydrogenation, methane

## Abstract

There is growing interest in specialized metabolites for fortification strategies in feed and/or as an antioxidant, anti-inflammatory and antimicrobial alternative for the containment of disorders/pathologies that can also badly impact human nutrition. In this context, the improvement of the diet of ruminant species with polyphenols and the influence of these compounds on animal performance, biohydrogenation processes, methanogenesis, and quality and quantity of milk have been extensively investigated through in vitro and in vivo studies. Often conflicting results emerge from a review of the literature of recent years. However, the data suggest pursuing a deepening of the role of phenols and polyphenols in ruminant feeding, paying greater attention to the chemistry of the single compound or to that of the mixture of compounds more commonly used for investigative purposes.

## 1. Introduction

Population growth and increased demand for food considerably augmented global livestock production, which was also massively affected through lifestyle changes. This led over time to an increase in diseases to which the animals are exposed, with impairment of the quality of the products brought to the consumers’ tables. Intensive husbandry systems for increasing milk yields in dairy cows showed, for instance, a negative impact on the animals’ health and welfare, which was related to an increase in the incidence of the major metabolic diseases. Thus, alternative feedstuffs are a need to be explored for improving animal production. Antibiotic growth promoters (AGPs) were broadly exploited in the livestock industry, with the increase in growth rate and efficiency of feed utilisation and the reduction of mortality and morbidity. However, their use/abuse was linked to an increase in antibiotic-resistant bacteria in livestock, so much so that in the European Union since 1 January 2006, antibiotics were deleted from the Community Register of authorised feed additives [1]. Indeed, although the ban defined infections’ increase in livestock with the decrease in animal production, the reduction of antibiotic resistance appeared to be averted, as well as the potential risk for humans. Interest thus turned to a plethora of other strategies involving feed enzymes, probiotics, prebiotics, phytonutrients or fatty acids [2,3], which could replace the drugs. Furthermore, environmental and health aspects play key roles in modern farming systems. Thus, the approach aimed at formulating animal feeds/supplements based on naturally occurring bioactive components with antimicrobial, antioxidant, anti-inflammatory and antitumor nutritional properties [4] found wider consensus. Plants, dried or in the form of their extracts, or also pure specialised compounds, appear to be valuable candidates as innovative alternatives to preserve livestock and their productivity, also taking into account the renewed awareness of consumers for natural healthy foods. Agro-industrial by-products (AIBPs) represent another interesting reservoir, and their use in small or large ruminants (i.e., goats, sheep, cows, buffalo), showed effects on rumen parameters (i.e., pH, gas, VFAs, ammonia, methane production), digestibility, and milk yield and chemical composition, with the main focus on the fatty acid profile [5,6,7].

Herein, the complex topic of plants and their polyphenolic specialised metabolites for animal health has been reviewed, focusing on the following different aspects: (1) chemical categorization of the main nutraceuticals used in livestock feed and different methods for their chemical identification; (2) effects of polyphenolic bioactive compounds on in vitro and in vivo rumen fermentation, with emphasis on methane emissions and the biohydrogenation (BH) process; (3) outcomes of bioactive (poly)phenol compounds on animal performance, milk production and composition.

## 2. Chemical Complexity of Bioactive Plant Compounds Used as Livestock Feed

**Figure 1:**
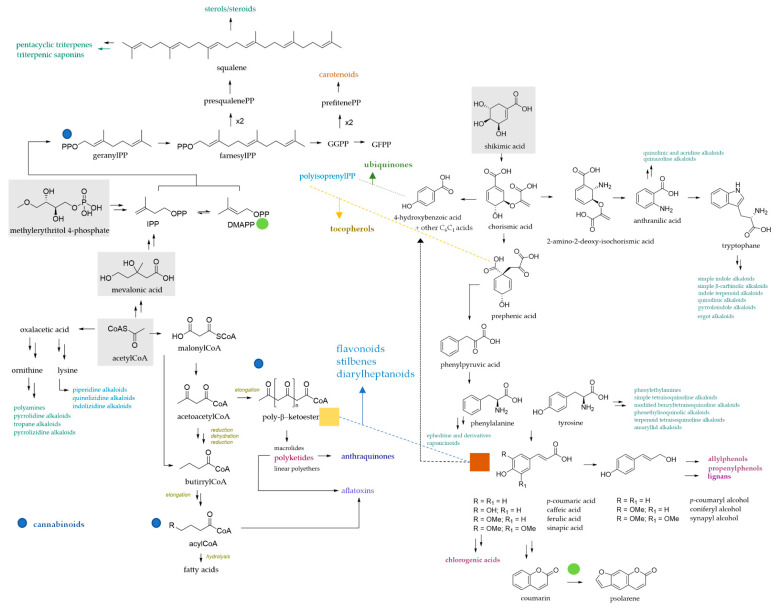
Schematic of the main biosynthetic pathways in plants to achieve phenols and polyphenols. Shikimic acid is the precursor of simple phenols and phenylpropanoids (hydroxycinnamic acid, coumarin lignans and lignins), which upon reacting with polyketides by acetate/malonate pathway favours the synthesis of flavonoids, stilbenes, flavolignans, and structurally related compounds. Terpenoid quinones are also produced when shikimic acid derivatives react with polyisoprenyl chains. The acetate pathway leads to fatty acid biosynthesis, by FAS enzyme, or poly-β-keto chains, which upon undergoing intramolecular reaction by polyketide synthase, leads to simple phenols, generally characterized by *meta*-substitution, or more complex compounds such as anthraquinones and aflatoxins. Three units of acetyl CoA combined by Claisen and aldol reactions lead to mevalonic acid, from which isopentenyl pyrophosphate (IPP) or dimethylallyl pyrophosphate (DMAPP) are synthetized. Methylerythritol 4-phosphate (MEP) is also a precursor of IPP/DMAPP. The two isoprene units are involved in the biosynthesis of terpenes, classified according to number of carbons. Essential oils (EOs), which are mixtures of low-molecular-weight terpenes, as well as triterpenic saponins, are deeply investigated for their pharmacological applications and different actions on ruminant health [9,10]. Alkaloids are biosynthesized by different precursors (the main classes from anthranilic acid, aromatic amino acids, ornithine and lysine are indicated). GGPP = geranylgeranyl diphosphate; GFPP = geranyl farnesyl diphosphate.

The diversity of bioactive compounds in plants has been known since ancient times, but only a few decades ago has interest been revived, thanks to the introduction of the nutraceutical concept. The term, an amalgamation of the words nutrition and pharmaceutical, was coined by Stephen De Felice, MD, founder and chairman of the Foundation for Innovation in Medicine (FIM), Cranford, NJ [8], to highlight the presence of compounds in foods able to positively modulate human health. Nutraceuticals provide a valuable tool in feed additives and can be classified following their mechanism of action, chemical nature, or feed source. Animal nutrition benefits from nutraceuticals, so much so that their production and use have increased during the last decade, even if non-ruminants and humans appear to be the main beneficiaries. Indeed, several studies highlight the health benefits deriving from the supplementation and/or integration of nutraceutical products as probiotics, prebiotics, dietary lipids, functional peptides and phytoextracts on both monogastrics and ruminants. Plant specialised metabolites, also known as plant secondary metabolites (PSMs), are of great interest in the nutraceutical overview and in animal feed restoration. These organic chemicals (Figure 1 [9,10]), biosynthesized in plants mainly for protection, growth, or stress response purposes [11], are from primary and intermediate metabolites through three well-defined pathways: shikimate, acetate and mevalonate/deoxyxylulose [12].

**Figure 2:**
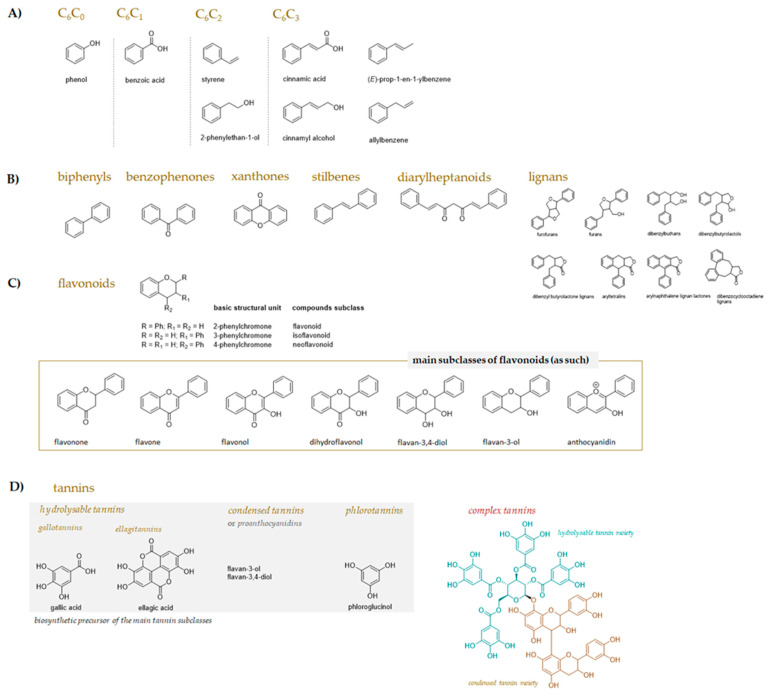
Main phenol and polyphenol skeletons. (**A**) Simple phenols (C_6_C_0_, C_6_C_2_ compounds) and phenolic acids (C_6_C_1_, C_6_C_3_ compounds); (**B**) non-flavonoidic polyphenols; (**C**) flavonoidic polyphenols and depiction of the skeleton of the main flavonoid subclasses; (**D**) tannins’ classification and depiction of the main precursor of each subclass.

Plants act as a reservoir of specialised metabolites; this is an intrinsic feature of the secondary metabolism complexity that makes plants a renewable source of bioactive compounds, differently employable for animal health and welfare. Phenols and polyphenols are the compounds that have attracted the most attention, and the investigation of their use in ruminant nutrition derives from their effects on rumen fermentation parameters, biohydrogenation, milk yield and composition, and also anti-inflammatory action [13,14]. These compounds broadly differ in their carbon skeleton and are biosynthetically from the shikimate and/or acetate pathway or an intertwining of the two. The shikimate route leads to essential aromatic amino acids, as well as phenylpropanoids, which could act as biosynthetic blocks for coumarins, lignans, or also stilbenes and flavonoids. These latter obtain part of their skeleton through the acetate/malonate pathway. Low-molecular-weight bioactive phenols (protocatechuic acid, vanillic acid, gallic acid, etc.) could be from the shikimate or acetate pathway, in accordance with a characteristic substitution pattern on the aromatic ring (Figure 2).

Ruminant nutrition mostly benefits from tannins and flavonoids [13]. These latter, with a C_6_-C_3_C_6_ skeleton and classified into six main sub-classes, are structurally composed of 15 carbons, with two aromatic rings connected by a three-carbon bridge, while tannins are a heterogeneous group of polyphenolic polymers distinguishable in three main groups: hydrolysable tannins (HTs), condensed tannins (CTs), and phlorotannins (PTs). HT and CT compounds are in terrestrial plants [15,16] and represent the classes of natural substances on which research has particularly focused in the last 10 years in terms of their effects on ruminal fermentation parameters, microbiota, biohydrogenation, and milk yield and composition.

### Multiple Approaches for (Poly)phenol Chemical Identification in Plant Extracts and Beyond

Spectrophotometric and colorimetric methods are broadly applied to quantify (poly)phenols in plant extracts, foods, or biological fluids, and are commonly based on the reaction of these substances, in their pure or mixed form, with a specific chemical probe. This yields a coloured adduct or complex, which can be measured spectrophotometrically at a defined wavelength [17]. Although colorimetric assays to quantify phenols, flavonoids, condensed/hydrolysable tannins and anthocyanins are very simple, inexpensive and fast approaches, they do not allow for reliable quantification of individual metabolites. However, these techniques have been widely used for a long time because they are useful to have a preliminary screening of the plant chemical composition, often further confirmed by antioxidant assays such as DPPH, ABTS, FRAP, and ORAC. Although this approach is very limiting, in most of the reviewed studies, the chemical analysis of plants and/or their extracts was colorimetrically performed without in-depth analyses of their chemical profiling. One of the main spectrophotometric assays for quantifying phenols is the Folin–Ciocalteu Reagent (FCR) method. This was applied for the first time to measure tyrosine [18], and then adapted for evaluating total phenols in wine [19]. The FCR method is based on the chemical reduction of a complex phosphomolybdic (H_3_PMo_12_O_40_)/phosphotungstic (H_3_PW_12_O_40_) acid to obtain a blue chromophore with the maximum of absorption at 760/765 nm [20]. The Folin–Ciocalteu test for total phenol content (TPC) has several advantages, such as simplicity and reproducibility, but also some drawbacks. In fact, the results could be distorted due to the presence of non-phenolic reducing compounds, such as ascorbic acid, sugars and some amino acids, which could lead to an overestimation of the data [21]. Instead, the most common method for assessing total flavonoid content (TFC) is based on the complexation, mainly through OH groups on C3, C5 and/or those on B-ring, with Al (III), with or without NaNO_2_. However, the method, in both its variants, lacks uniformity of reaction, resulting in an unsuitable identification of flavonoids in unknown samples [22]. Total anthocyanins (TAs), taking into account their unique structure, are commonly estimated through pH variations, which lead to structural modifications of the anthocyanin chromophores. Different techniques have been employed for tannin identification, beyond colorimetric assays [23]. The butanol-HCl assay is used for quantifying condensed tannins; it is based on the cleavage of interflavanic bonds to achieve flavan-3-ols, which undergo auto-oxidative reaction, providing a mixture with maximum absorbance at 550 nm. Proanthocyanidin content is also estimated by vanillin assay [24] or using 4-(dimethylamino)-cinnamaldehyde (DMACA). The potassium iodate (KIO_3_) method allows hydrolysable tannins to be quantified [25]. Two other methods are the rhodanine assay and the sodium nitrite method [26]. Precipitation methods employ proteins, such as bovine serum albumin (BSA), or polymers such as polyethylene glycol (PEG), polyvinylpyrrolidone (PVP) and polyvinylpolypyrrolidone (PVPP). In particular, the BSA method, involving the formation of both hydrogen bonds and hydrophobic interactions, favours the formation of insoluble tannin–protein complexes. The tannin–protein complexes, dissolved in alkaline sodium dodecyl sulphate-triethanolamine, are able to react with FeCl_3_ so much so that a violet complex absorbing at 510 nm is obtained [21]. Indeed, due to the complexity of a living organism and the biotic and abiotic factors that can induce continuous changes in the qualitative and quantitative composition of extractable polyphenols, the use of the colorimetric assay is advisable only as a preliminary approach, and is not useful to fully clarify plant/extract chemical composition and its effects in a biological system. In particular, NMR, UV/Vis and MS-based techniques, used alone or coupled with separative techniques (generally, LC, GC, CE or SFC) are the most employed for carrying out metabolomics studies [21]. Mass spectrometry, hyphenated with LC or GC, is by choice used for obtaining insight into this class of compounds. Indeed, the low volatility of phenolic compounds restrains the applicability of GC-MS techniques without a proper derivatization, allowing MS-coupled high performance liquid chromatography (HPLC or UHPLC) to actually represent the most effective technique for analysing phenols in mixture. Reversed-phase (RP) HPLC is commonly used for separative purposes. In this context, the length of hydrophobic alkyl chains (C8, C12 and C18) plays a key role, as well as the choice of eluents such as methanol or acetonitrile to be combined with water in gradient and/or isocratic chromatographic runs [27]. MS ionisation improvement, or peak tailing effect reduction, requires the addition of low amounts of acids (e.g., acetic, formic or phosphoric acids) as mandatory. Undoubtedly, compared to colorimetric assays, an approach that joins advanced analytics technologies has higher costs but offers more reliable data. However, LC-MS analysis can undergo matrix effects (MEs) that suppress or enhance the ionisation, providing subsequent quantification errors. Even compared to NMR, the MS tool is much more sensitive, fast and less expensive. To overcome MEs, fractionation through chromatographic and extractive techniques is broadly suggested. It is certain that although mainly HR-MS tools are fundamental to detect novel compounds, NMR techniques provide incontrovertible results in terms of chemical identification of new compounds. Indeed, to better throw a spear in favour of MS, the wide spectrum of analysers (simple quadrupole (Q), triple quadrupole (QqQ), quadrupole time of flight (Q-TOF), and orbitrap-based hybrid mass spectrometry (LTQ-orbitrap)) currently allows various possibilities of analysis that take mass spectrometry from a simple instrument for the recognition of molecular weight to a useful identification approach. Nowadays, electrospray ionisation (ESI) in negative ion mode has emerged as one of the most sensitive analytical methods to acquire structural information on phenol and polyphenol compounds [28]. Where the detection in mass spectrometry can benefit from that in UV-Vis spectrophotometry, which exploits well the arenic nature of these compounds, is that the study in mixtures is exhaustive in terms of qualitative and quantitative knowledge [29].

Despite the advantages and accuracy of these analytical techniques (useful for the identification and quantification of phenols and polyphenols in food, plant matrix and/or biological fluids), an analysis of the literature data on the specific correlations between polyphenols and their effects on ruminants found that only a few authors report the chemical characterization of the plant extracts under study, by LC-UV or MS and GC-MS [30,31,32,33].

Most of the authors provide bioactivity data in reference to the test of an extract, classified as “enriched with polyphenols” on the basis of the above-mentioned colorimetric assays. Indeed, especially when evaluating the biological behaviour of a mixture, knowing the qualitative and quantitative composition is of indispensable importance, where even small variations in the concentration of a substance can also significantly influence the bioactivity. Innovative approaches to the study of the impact of plant-specialized metabolites on rumen properties need to be further examined through a metabolomic analysis, which has already been applied to rumen fluid. The latter, possibly subjected to liquid–liquid extraction (LLE), could be analyzed by NMR spectroscopy, GC-MS and/or LC tandem mass spectrometry. The several and few “bibliomic” studies led to the creation of a Bovine Rumen Database (BRBD) (http://www.ruendb.ca) [34,35], which is a web-accessible resource containing several identified and quantified rumen metabolites [36]. 

## 3. Focus on Rumen Fermentation: A Key Step to Unravel Polyphenol Efficacy

The digestive system of ruminants, such as cattle, equips them to efficiently use plants, including grass, fodder and agricultural by-products that cannot be digested by monogastric animals. Ruminants have a stomach with four compartments (rumen, reticulum, omasum, and abomasum) characterized by a normal microbiota, which differs in composition and functions. For its population of microorganisms, the reticulo-rumen is the main digestive tract, responsible for digestion and the utilization of proteins, carbohydrates and fatty acids. After this tract, the digesta flows into the omasum, where water reabsorption and particle size reduction occur. Finally, it passes into the abomasum (true stomach), which secretes the digestive enzyme that prepares the digestive system for absorption in the small intestine, where the absorption of proteins, vitamins, simple carbohydrates, fatty acids and amino acids takes place. Anything that is not absorbed will pass through the large intestine and will be excreted via the stool. 

A deeper understanding of the chemical and nutritional composition of foods supplied to ruminants has been required due to the growing interest in animal feed to ensure the availability of essential nutrients. In fact, it is known that the digestibility of a food is closely related to its chemical composition. The dry matter (DM) of food is conveniently divided into organic and inorganic material, of which the main component for ruminant food is carbohydrates. Traditional food parameters are moisture, ash, crude protein (CP), ethereal extract (EE), crude fibre (CF) and nitrogen-free extracts (NFEs). Furthermore, modern methods of ruminant feed analysis attempt to distinguish between cell content fractions and cell walls, which, after treatment of fodder with a neutral and healthy detergent solution, remain as a residue called neutral detergent fibber (NDF). NDF provides an estimate of cellulose, hemicellulose and lignin levels negatively correlated with voluntary feed intake. The cell wall fraction can be further divided into acid detergent fibre (ADF), which represents cellulose and lignin, and acid detergent lignin (ADL), which represents lignin only. The cell content is almost completely digested, while the digestibility of the cell walls is much more variable and depends on the degree of lignification (ADL) [37]. While new technologies offer insight into the chemical composition of foods, rumen digestion mechanisms are incredibly complicated. The data acquired often do not reveal information on how the nutrients are actually used by the animal. Although in vivo research is the most effective way to characterize the dynamics of digestion in ruminants, in vitro rumen fermentation experiments recreate, albeit in a simplified way, what happens in the rumen, providing further information. In vitro rumen fermentation techniques can provide information on dry matter digestibility, crude protein, gas production and fermentation rate, as well as information on fermentation of end products such as methane, pH, total volatile fatty acids (e.g., acetate, butyrate, propionate, BFCA) and ammonia production [33,38,39].

**Figure 3:**
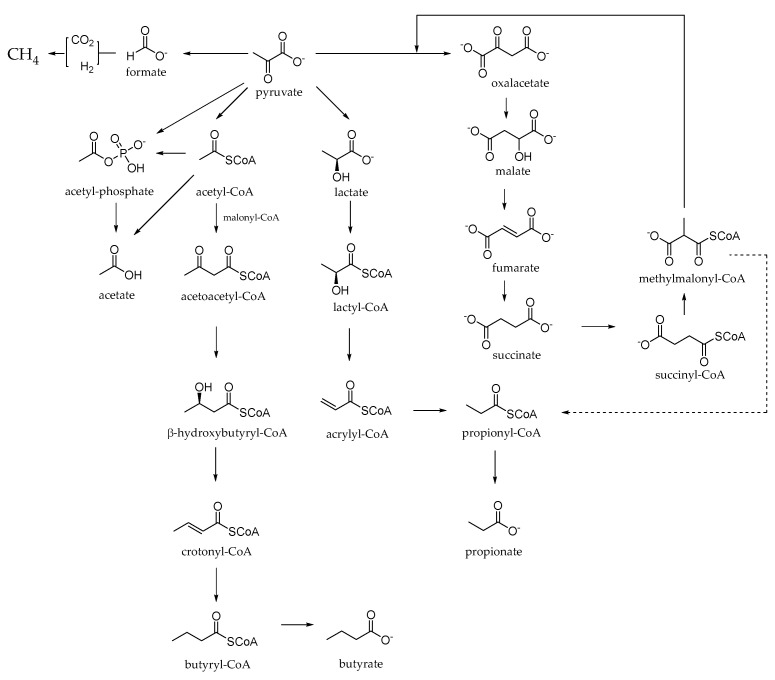
Main volatile fatty acids biosynthesized by microbes in ruminal fluid from pyruvate.

Since the rumen serves as the primary site for microbial fermentation of ingested plant material, rumen fermentation is the process in which carbohydrates are converted into simple sugars. Microbes use some of these sugars to grow and multiply, while cattle use the rest as their main source of energy. Pyruvate is the key intermediate of this process and is related to the main end products of digestion of rumen carbohydrates such as acetic, propionic and butyric acids (mainly absorbed through the rumen wall), heat and gas (e.g., carbon dioxide and methane), lost by belching (Figure 3). Excessive decompensation of volatile fatty acids (VFA) could cause subacute ruminal acidosis (SARA), a metabolic disease responsible for laminitis, diarrhoea, damage to the rumen mucosa, rumenitis and liver abscesses in dairy cows [40]. In the large intestine, there is a second stage of microbial digestion, and the volatile fatty acids produced here are absorbed, while the microbial cells are excreted with the undigested food components in the faeces [37]. The volatile fatty acids of the rumen can be involved in different metabolic pathways, so their modulation mechanisms, through the daily diet, can lead to macroscopic effects in the animal. For example, acetate is used as the primary energy source in the lipogenic process, while propionate is a precursor of gluconeogenesis, and butyrate is mainly metabolized into D-3-hydroxybutyrate. Furthermore, acetate and propionate play a fundamental role in establishing an internal hydrogen balance by regulating the production of rumen methane; acetate biosynthesis produces metabolic hydrogen, the main substate of methanogenesis, which is consumed by the formation of propionate from pyruvate [41]. Other volatile fatty acids, called branched fatty acids (BCFA), are also formed in the rumen by deamination of amino acids such as isobutyric acid from valine, valeric acid from proline, 2-methyl butyric acid from isoleucine and 3-methyl butyric acid from leucine [37]. In fact, food proteins are hydrolyzed into peptides and amino acids by rumen microorganisms, and some amino acids are further degraded into organic acids, ammonia and carbon dioxide. In this case, ammonia is further used to synthesize microbial proteins, becoming a rumen predictor of the efficiency of the conversion of dietary N to microbial N [42]. Furthermore, dietary proteins are not the only contributor to the ammonia pool in the rumen, considering that the nitrogen source can be derived from amino acids, amides, amines or inorganic compounds such as nitrates. 

Since the ecosystem of the rumen microbiota is diverse and consists of anaerobic bacteria, protozoa, viruses and fungi [43] with different functions [44], PSMs with antimicrobial efficacy could be able to modulate rumen fermentation, increasing digestion and metabolism of nutrients, and thus VFA production, with further methanogenesis suppression. Indeed, multiple factors, including the diet, should be considered to test the effect of pure polyphenol or plant extracts on rumen fermentation, methane emission and microbial composition [45].

### 3.1. Rumen Microbiota Composition

The bacterial community depends on the animal species, the type and chemical composition and/or frequency of the diet. In all ruminants, bacteria belong to the phyla of Firmicutes, Bacteroidetes, Proteobacteria, Fibrobacteres and, in a small number, to Tenericutes and Actinobacteria [46]. Their classification generally takes into account the relative feed substrates so that cellulolytic, amylolytic, proteolytic, lipolytic, methanogenic, saccharolytic, tanninolytic, pectinolytic, ureolytic, acetogenic and acid users can be distinguished. The three major cellulolytic bacteria, found in cows and other ruminants, are *Fibrobacter succinogenese* (Gram-), *Ruminococcus flavefaciens* (Gram+) and *Ruminococcus albus* (Gram variable) [47], whose fermentation end products mainly consist of acetate, butyrate, propionate, and CO_2_. The most important pectinolytic species in bovine metabolism are *Lachnospira multiparus*, able to reduce pectin to oligogalacturonides providing a great acetate amount, *Prevotella ruminicola* and *Butyrivibrio fibrisolvens* [48]. Proteolytic bacteria (i.e., *Clostridium*, Bacilli and Proteobacteria) break down proteins into smaller peptides; proteolytic enzymes, such as urease, able to degrade urea in carbon dioxide and ammonia, define their action. *Anaerovibrio lipolytica* and *Butyrivibrio fibrisolvens* are lipolytic bacteria, whereas rumen methanogenic archaea bacteria include Methanococcales, Methanomicrobiales, Methanosarcinales, Methanopyrales and Methanobacteriales. These latter consist of the four genera *Methanobacterium*, *Methanobrevibacter*, *Methanosphaera* and *Methanothermobacter* [49], and constitute the bulk portion of the methanogen community in the majority of ruminants [50]. In recent years, the research on this group of microbes has improved the understanding of how the possible diet intervention and relationship with other microbes can modify livestock CH_4_ emissions [51]. According to rumen protozoa, this microbial class was first discovered by Gruby and Delafond [52] and divided into two main groups: “holotrics” and “entodiniomorphs”; the first group uses soluble sugar, while the second one controls the rate of carbohydrate fermentation when the amount of soluble carbohydrate increases in the diet. In particular, entodiniomorphid ciliates act in controlling starch digestion by engulfing starch granules [53].

Although rumen anaerobic fungi are a minor part of the biomass of rumen microorganisms, they are important because they are involved in the degradation of lignocellulosic components. This group of microbes is mainly found in the reticulo-rumen, and feed is acquired through a system of flagella. In particular, they are able to produce different types of enzymes, belonging to the family of hydrolases such as cellulase, esterase, glucosidase, or xylanase, the variety of which depends on their phylogenetic origin and, above all, on their rhizoidal structure. In this way, they can penetrate the plant cell walls and colonise the plant structure. In fact, rumen fungi are important when ruminants consume lignified substrates. In cows, different fungi have been identified, such as *Pyromices communis*, *Neocallimastix frontalis*, *Neocallimastix hurleyensis, Anaeromyces elegans, Anaeromyces mucronatus, Orpinomyces joyonii, Orpinomyces intercalaris,* and, also in buffalo, *Orpinomyces bovis, Pyromices communis* and *Cyllamyces aberensis* [54]. 

Since the composition of the rumen microbiota, the efficacy in feed degradation or methane production and the BH process, as well as the composition of milk and/ or meat could be influenced by the diet [13,55,56], the evaluation of the impact of non-nutrient substances such as plant-specialised metabolites cannot be ignored. The effects of polyphenols on rumen fermentation parameters, methane production and VFA are summarised below, based on a review of the literature of recent years.

### 3.2. Effect of Polyphenols on Rumen Fermentation Parameters

Methane is one of the main greenhouse gases (GHG), and ruminants are the main producers, with a value of between 250 and 500 litres of methane per day, so much so that, in particular, cattle are considered to highly impact global warming [57]. However, CH_4_ accounts for less energy than the ruminants’ feed intake. It arises from the fermentation of the feed in the rumen (87–90%) and in the large intestine (10–13%) of ruminants by the action of methanogenic archaea. Methanogens reduce CO_2_ to CH_4_ by using H_2_ during the last stage of microbial rumen fermentation. In particular, primary digestive microorganisms hydrolyse proteins, starch and cell wall polymers into simple sugars, which are then fermented into VFA, hydrogen and carbon dioxide [58]. 

**Figure 4:**
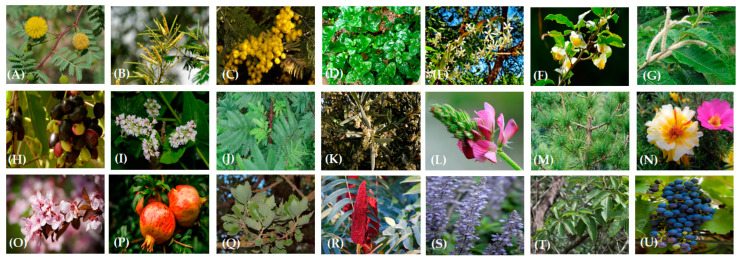
Examples of plant sources whose polyphenolic extracts are of broad interest in investigations focused on their ability to affect rumen fermentation parameters. (**A**) *Acacia arabica*; (**B**) *Acacia catechu*; (**C**) *Acacia dealbata*; (**D**) *Alternanthera sissoo;* (**E**) *Azadirachta indica; (***F**) *Camellia sinensis;* (**G**) *Castanea mollissima;* (**H**) *Eugenia jambolana;* (**I**) *Fagopyrum esculentum;* (**J**) *Mimosa tenuiflora;* (**K**) *Olea europaea;* (**L**) *Onobrychis viciifolia;* (**M**) *Pinus radiata;* (**N**) *Portulaca oleracea;* (**O**) *Prunus cerasifera;* (**P**) *Punica granatum;* (**Q**) *Quercus ithaburensis* subsp. *macrolepis;* (**R**) *Rhus typhina;* (**S**) *Salvia officinalis;* (**T**) *Schinopsis balansae;* (**U**) *Vitis vinifera*.

In recent years, various strategies have been used to reduce methane emissions from livestock, and a revival of plant-based nutrition, as it is natural or enriched with PSM extracts from different plant sources (Figure 4), appears to be among the most promising. 

Polyphenols, mainly tannins, act directly or indirectly. The indirect action is due to their lowering degradability effects on plant material [59,60]. The direct effect occurs when plants or extracts inhibit methanogenic microbes [61]. Furthermore, plant constituents can reduce the accessibility of methanogens to H_2_ by favouring the increase in propionate, a gluconeogenic precursor [62], whose fermentation path, unlike other VFAs, does not lead to hydrogen release. The modulatory effects of flavonoids on methane emission are inscribed in their structure and in the variable degree of substitution, especially in terms of phenolic functions. The antibacterial action appeared to be influenced by the type of substituents on the aromatic rings [63]. 

**Figure 5:**
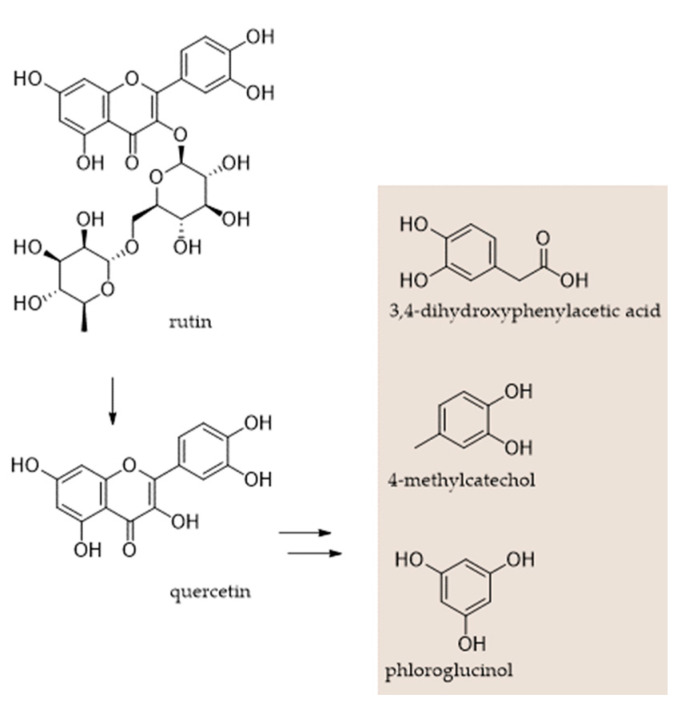
Quercetin, and its rutinoside (rutin), as well as the metabolite 3,4-dihydroxyphenylacetic acid, 4-methylcatechol, and phloroglucinol. Berger et al. [70] showed that when incubated in vitro with rumen, quercetin disappeared, and 3,4-dihydroxyphenylacetic acid rapidly increased, followed by 4-methylcatechol. Both quercetin and rutin did not affect in vitro methanogenesis.

An alcoholic extract from *Portulaca oleracea*, a herbaceous plant belonging to the Portulacaceae family [64], consisting mainly of derivatives of kaempferol, quercetin, apigenin and luteolin, has been found to increase the production of total VFAs, in particular acetate and propionate, and reduce methane production by 9.44%, with a halving of the methanogen population compared to the control. Methanol extraction of *Pinus radiata* bark has also led to an extract rich in flavonoids (luteolin, quercetin, taxifolin, catechin) and with small quantities of stilbenes and phenolic acids, capable of reducing, in a dose-dependent manner, significantly after 6h, CH_4_ production and increasing the total VFAs after 24 h of incubation [65]. A flavonoid extract from Brazilian spinach (*Alternanthera sissoo*), integrated with 40 mg of substrate, reduced the population of protozoa to a value of 1.79 cells/mL, also causing an increase in propionic acid and a massive reduction in the production of CH_4_, which ranged from 20.22 mmol/L to 13.48 mmol/L [66]. The structure of the aglycone and the degree of glycosylation seem to play a fundamental role, since by testing different flavonoids at a dose of 4.5% of the substrate (dry matter basis), the methane emissions were decreased in descending order as follows: myricetin ≥ kaempferol ≥ quercetin ≥ naringin ≥ rutin ≥ catechin [67]. The data are not all positive, and often the use of a single molecule to be proposed as an additive in feed has failed to render the desired effects. This is the case with metabolomic studies aimed at defining the action of catechin in rumen fluid [68], which seems to be involved in the sequestration of three H_2_ molecules, or of rutin, which, when supplemented at a dose of 100 mg/kg to dietary cows, did not favour any decrease in methane emission [69]. In vitro methanogenesis investigation with rutin and quercetin also had negative effects [70] (Figure 5), whereas in vivo rutin supplementation led to an increase in total VFAs without a significant variation in the rumen protozoa [71]. It is certain that the variables involved, including the compound dose, rarely appear similar. 

Although, it is not possible to make general assumptions, probably the degree of oxidation of the flavonoid nucleus plays a role. In fact, it was observed that luteolin 7-*O*-glucoside was more efficacious than the flavonols quercetin and isoquercitrin, as well as of catechin derivatives in reducing CH_4_ emissions, exerting an activity similar to that exerted by tannic acid but without a shift in the acetate/propionate ratio or reduction of protozoal counts [72]. The total VFAs could be also unaffected by all the subclasses of flavonoids, whereas they are capable of varying the acetate/propionate ratio, increasing propionate and thus depressing CH_4_ emissions [73]. 

The plant extract-induced decrease in total gas and methane could be justified by (1) a reduction in protozoa and/or the methanogen population, associated with protozoa surface, (2) direct inhibition of methanogen population, (3) changes in VFA profiles, and (4) inhibition of fibrinolytic enzyme activity and feed digestibility. Kim et al. [74] also reported that total VFAs increased or decreased based on considered incubation times, while the methane emission was significantly lower than control. This effect could be due to the ability of flavonoid-rich extracts to reduce more than 60% of the protozoa population. In particular, it was observed that *Fibrobacter succinogenes* increased in its diversity, while *Ruminococcus albus* and *Ruminococcus flavefaciens* populations diminished. Therefore, even if the real chemical composition of the plant extracts investigated was not known, the amount of total phenols or flavonoids is used as a tool (not exhaustive and limited) to predict the role of these compounds, and it has often been observed that the content of the different classes of compounds provided a great variability in terms of expression of the TPC and TFC value. In this occurrence, *Olea europaea* L. leaf (OLs), whose TPC and TFC were found equal to 34.79 ± 2.72 mg catechin/g extract and 5.91 ± 0.24 mg quercetin/g extract, reduced CH_4_ emission significantly after 12 h, maintaining the acetate/propionate ratio [30]. It is not clear what reason drives researchers to express TPC content using a flavanol and not also a simple phenol, such as gallic acid, to express TPC content. Furthermore, it is noteworthy that previous studies on olive leaf extracts consisting of oleuropein, tyrosol, hydroxytyrosol, caffeic acid, gallic acid, syringic acid and luteolin, also observed a decrease in both methane production and VFAs [75], increasing mainly rumen acetate. This effect was also found using a propolis phenolic extract dosed at 16.9 and 33.9 mg/d. The increase in acetate was positively linked to fatty acid synthesis [76]. It is possible that the flavonoid identity favours acetate or propionate production, as it was often observed, as in the case of supplementation with flavonoids from *Candida tropicalis*, that propionate was augmented among rumen fermentation products [77]. 

Condensed and hydrolysable tannins appeared to be the most active, and their activity was far greater than those explained by their monomers, which appeared to have no effect on the in vivo CH_4_/total gas ratio, in contrast to in vitro findings [68]. The main goal of CTs is, beyond modulation of the short-chain fatty acid (SCFA) profile, the inhibition of methanogenesis. A recent screening on 21 medicinal and aromatic plants, selected based on their total CT and HT contents [78], suggested that leaves with the lowest CT content (138 g/kg DM) and highest amount of total phenol (108 g/kg DM), such as those of neem (*Azadirachta indica* A. Juss), suppressed CH_4_ emission by 61.5%, potentially through a direct action on rumen archaea [79]. 

Biomass materials represent a good source of these compounds and are deeply investigated for tannin achievement. Hydroalcoholic extracts of chestnut (*Castanea* spp.), sumac (*Rhus typhina*), mimosa (*Mimosa tenuiflora*) and quebracho (*Schinopsis balansae*), all supplemented at the dose level of 1 mg/mL, inhibited methanogens, whereas they exerted a different dose-dependent behaviour against *Fibrobacter succinogenes*, *Ruminococcus flavefaciens* and anaerobic fungi [80]. Quebracho and chestnut tannin supplementations, although shown not to affect VFA levels so far, are widely accepted. Indeed, the biological response is in the dose level considered, so much so that a decrease in total VFAs was observed when quebracho tannin extract was supplemented (on a dry matter (DM) basis) at 2% or 3% [81]. Recently, a hydroalcoholic extract from involucres of *Castanea mollissima* Blume, the Chinese chestnut, supplemented at 0.2%, significantly reduced the CH_4_ yield during in vitro rumen fermentation [82]. Other sources of anti-methanogenic tannins are found to be in the acorn cups of valonia oak (*Quercus ithaburensis* subsp. *Macrolepis*) and in Myrobalan plum (*Prunus cerasifera*) [31], whose action appeared to be due to chebulic ellagitannins (ChET), which are plant-derived polyphenols containing chebulic acid subunits [83], as well as green tea (*Camellia sinensis*) and grape seed (*Vitis vinifera*). This latter was proved to improve CH_4_ emission reduction at different dose levels (0.2, 0.4, 0.6 and 0.8%) in a diet soy oil-enriched at 2.5%, while ruminal VFA concentration was negatively impacted when CTs in grape seed extract were more than 0.4% [84]. Grape marc also was able to decrease (by 20%) CH_4_ emissions, when supplemented in the form of pelleted grape marc or ensiled grape marc [85]. The contrasting effect could be minimised through the full determination of tannins (or other compounds of interest) identified in the extract, also taking into account the plant organ, its harvesting time, the geographical area of collection, the plant phenological stages and other biotic and abiotic factors. This is in line with the different CT content and polyphenols in the perennial legume sainfoin (*Onobrychis viciifolia* Scop.), collected at different phenological stages, to which a variable CH_4_ emission appeared to correspond [86], or also the observation that the flowers, and not leaves, of buckwheat (*Fagopyrum esculentum* Moench) suppressed methane per unit of total gas by 10%, in a way comparable to that of rutin when supplemented at 50 mg. Recently, an interesting investigation on the seasonality effects of *Acacia* spp. on nutrient content, polyphenols and antioxidant activity showed that *A. mearnsii* had a nutritional and nutraceutical value higher than that of *A. dealbata* leaf-meals [87]. This finding was in line with a previous report on the ability of *Acacia mearnsii* to positively impact animal production, showing an effect on methanogenesis when used at the level of 30 g/kg (equivalent to 15 g total tannin/kg as analysed) [88]. Furthermore, testing increasing levels (0, 5, 10, 15, and 20 g/kg of diet DM) of *A. mearnsii* CTs in the diets of Jersey steers, it was found that the ruminal protozoa population was not affected by CTs, while protein digestibility, ruminal pH, and acetate proportion were decreased [89]. An in vivo study using *A. mearnsii* extract at 100 g tannin extract cow/day feed supplementation observed that it was less active than the polyether antibiotic monensin in reducing CH_4_ emissions or increasing total VFAs [90,91]. *Acacia* spp. were evaluated for their effects also in combination with other species, as in the case of the CPRE extract, which was from *A. arabica* (burk), *A. catechu* (burk), *Punica granatum* (peel) and *Eugenia jambolana* (seeds), which was tested in vivo and in vitro in murrah male buffalo. CPRE mitigated methane production in vitro and in buffaloes, and the effect was related to the content in flavonoids, tannins and saponins, able to reduce methane production by inhibiting methanogens and protozoa-related methanogens or by lowering hydrogen production from fibre degradation [92]. 

It is certain that the mechanism by which CTs mediate the reduction of CH_4_ emission is unknown, and potentially the ability of CTs to form complexes with dietary proteins and carbohydrates in the rumen plays a key role, but inefficient tannin quantitation could result in non-unique effects shown by the different plant extracts. 

**Figure 6:**
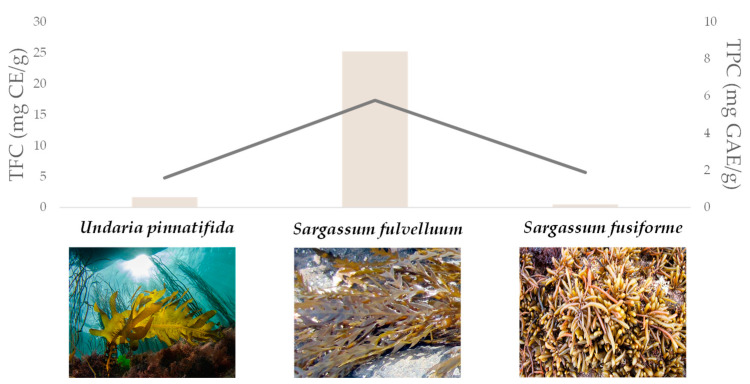
Algal species affecting rumen fermentation parameters TPC and TFC [96]. TPC = total phenol content; TFC = total flavonoid content.

Tannins could co-exist with other compounds, and these latter could diversely influence, in a synergistic or antagonistic way, the response observed. Thus, the extraction method employed could be a determinant, as suggested by data on *Vaccinium vitis idaea* tannin extract, which was first shown to reduce by 8.5% the CH_4_ emissions, and the protozoa population by 35%, without affecting methanogen counts [93]. Furthermore, using the rumen simulation technique (RUSITEC), a well-established semicontinuous in vitro model for investigating ruminal fermentation, it was found that *Vaccinium vitis idaea* decreased methane by 21.9% [94]. It appears clear that methane emissions are strongly affected by microbiome features, and although data are not consistent, low and high methane phenotypes provide the observed variability. Other interesting examples are from *Salvia officinalis*, belonging to the Lamiaceae family, broadly investigated for its benefits for human and animal health. PSMs in *S. officinalis* consist of phenols such as rosmarinic acid and salvianolic acids, but no attention was paid to these substances when sage extracts were analysed for their effects on rumen cellulolytic bacteria populations, digestibility and methane emission. In fact, it was reported that 40 mg and 100 mg of sage extract, corresponding to the tannin input of 51.2 g and 128.0 g per kg dry matter, reduced methane and also VFAs, considered in their totality or as a single compound [95], whereas at a dose equal to 4 mg, the sage extract exerted an activity only on microbes, being able to stimulate some protozoan species. Three brown seaweed extracts (*Undaria pinnatifida*, *Sargassum fusiforme* and *Sargassum fulvelluum*) decreased methane gradually after 12 h and 24 h of incubation; regarding the microbial counts, there was no significant change in the absolute value of total bacteria (Figure 6). The populations of both ciliate protozoa and fungi were significantly lower for *Sargassum fusiforme* and *Sargassum fulvelluum*, but none of the seaweed extracts decreased massively methanogenic archaea [96]. 

An analysis of the most recent literature strongly suggests that polyphenolic compounds exert different effects, but the data are often conflicting. This is particularly due to the comparison of extracts that, although prepared from the same plant, may have different chemical compositions. The extraction technique massively affects the chemistry of an extract, and the use of different extraction techniques or even just different extractants can lead to extracts that are not comparable in terms of bioactivity. 

## 4. Biohydrogenation (BH) Process and Effects of Polyphenols on Lipidic Profile

Ruminant-derived food is an important source of fatty acids for the human diet. 

**Figure 7:**
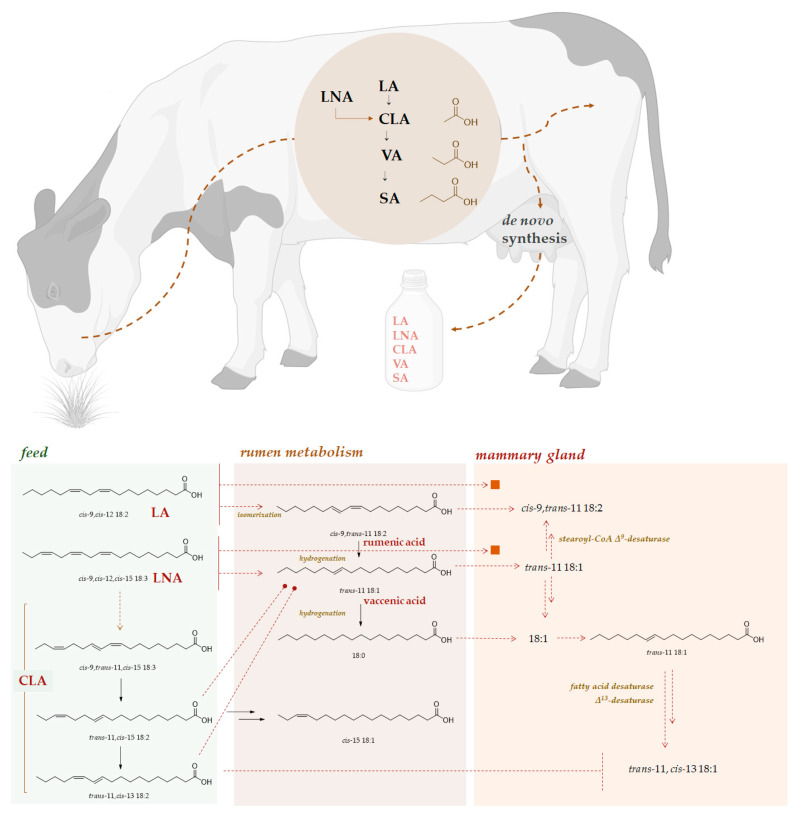
Schematic ruminal biohydrogenation process of free unsaturated fatty acids in rumen and milk fat profile influenced by de novo synthesis in the mammary gland and by mobilization of adipose tissue. Figure created with BioRender.com. LNA, linolenic acid; LA, linoleic acid; CLA, conjugated linoleic acid; VA, vaccenic acid; SA, stearic acid.

Biotransformation processes promote unique FA synthesis. In fact, although considering milk from different species, it can be stated that the diet defines its FA content, in ruminants a key role is attributable to rumen bacteria BH [97]. Ruminant milk FAs derived from intestinal absorption of dietary and microbial FAs and an amount < 10% by lipolysis and mobilisation of body fat (Figure 7). 

FAs in ruminants’ diet mainly include essential polyunsaturated fatty acids (PUFAs) *n*-3- or *n*-6-type (α-linolenic and linoleic acids), whose intake is from forages, cereals or oil seeds, long chain PUFAs from marine products and monounsaturated fatty acids (MUFAs). Commonly ingested as triacylglycerols, saponifiable lipids undergo hydrolysis and BH by rumen microbiota. The hydrolysis cleaves ester bonds between glycerol and FAs. Microbial lipases are prompt to hydrolyse, which is thanks to *Anaerovibrio lypolitica*, which, diversely from *Butyrivibrio-like* species such as *Butyrivibrio fibrisolvens*, is not able to hydrolyse phospho- and galactolipids. Lipase activity occurs in ciliated protozoa, although it has a minor contribution compared to bacteria, but it is unclear whether it can occur in fungi [98]. After hydrolysis, the transformation of dietary fatty acids into saturated fatty acids (SFA; 18:0) and conjugated linoleic acids (CLA) is catalysed by rumen microorganisms. Two groups of bacteria are involved: group A bacteria reduce 18:2 (*n*-6) and 18:3 (*n*-3) to *trans*-11 18:1, while group B bacteria add H_2_ to 18-carbon PUFAs, converting them to stearic acid [99]. Bacteria, protozoa and fungi are actors in the BH process. The first ones are the main players, whereas protozoa are reported to be involved in CLAs and vaccenic acid production [100], and fungi (above all, those of *Orpinomyces* genus) are observed to biohydrogenate linoleic acid (LA) to vaccenic acid, though they are slower than bacteria [101]. 

The BH process utilizes as substrates α-linolenic acid (LNA; *cis-*9,*cis-*12,*cis*-15 18:3) and LA (*cis-*9, *cis*-12 18:2). In particular, beyond different biosynthesised isomers, LA is favourably converted first into rumenic acid (*cis-*9,*trans*-11 18:2), then into vaccenic acid (*trans*-11 18:1) and finally into stearic acid (18:0). Analogously, LNA is metabolized through a more complicated pathway, whose key intermediate is a conjugated triene, *cis*-9,*trans*-11,*cis*-15 18:3. The great part of BH products is adsorbed in the gut and secreted into milk, whereas another part is Δ^9^-desaturable substrate in mammary glands, thus providing other derivatives, secretable into milk [102].

Dietary manipulation, through lipid supplementation increase or specialized metabolite involvement, affects BH, altering rumen microbiota activity with changes in milk and meat FA composition.

Polyphenols are able to reduce or modulate the BH process by modifying the rumen bacterial community. This is in line with the decrease in stearic acid synthesis, as previously reported [13,103,104]. Indeed, different effect could be ascribed to CTs and HTs. Specifically, CTs showed a BH inhibitory effect, whilst HTs appeared to prevent essential FA BH. 

The modulating efficacy of polyphenols with detected disappearance of BH products was observed by Jayanegara et al. [105], who highlighted that HTs from leaves of *Castanea sativa* L. were positively correlated to *cis*-9,*trans*-11 18:2 occurrence. Quinones, formed following phenol oxidation by polyphenol oxidase (PPO), indirectly affect the BH process, such that their interaction with lipolytic enzymes provides a physical barrier that slows down the activity of rumen bacterial lipases. Since PUFAs are not biohydrogenated, they have the possibility of being preserved in products for human consumption, with all the benefits commonly ascribed to them [106,107,108]. 

The content in tannic polyphenols from *Vicia sativa* and *Trifolium incarnatum* harvested at vegetative and reproductive phenological stages was observed to affect the ruminal microbial population and microbial activity [107]. This could be due to the chemical features of tannins. Furthermore, in accordance with the view that one polyphenol is not worth another, when grass silage was replaced with red clover silage in the diet of lactating cows, it was observed that the higher PPO activity of red clover silage induced an increase in 18:2 *n*-6 and 18:3 *n*-3 and non-esterified *cis*-18:1 (Δ^11,12^) and *trans*-18:1 (Δ^12,16^), and a reduction in *trans*-10, *cis*-12 CLA, *trans*-11,*cis*-13 CLA, and *trans*-9,*trans*-11 CLA in the omasum [108].

Polyphenols are suggested to mediate the depression of protozoa, which are the major hydrogen producers [104]. The hydrogen storage reduction could lead to competition between BH and methanogenesis, and the supplementation with tannin extract of *Acacia mearnsii* led to an increase in *trans*-11 18:1, inhibiting the reduction to stearic acid [104].

The role of dietary tannins in influencing in vitro FA BH was also related to the decrease in iso- and odd chain FAs, while vaccenic acid increased, thus also augmenting CLAs after 120 h of treatment. This was in line with the ability of tannins to interfere with microbial proliferation after a long period of fermentation [109]. However, contrasting data are from quebracho condensed tannin, which, dosed at 100g/kg on diet DM, did not show an effect on the BH of C18 unsaturated fatty acids [110], while protecting flaxseed PUFAs from BH in the rumen [111]. The role of tannins’ antimicrobial property in BH is not clear [112], although *trans-*10 18:1, *trans-*11 18:1 (vaccenic acid), *cis*-9, *cis*-12 18:2 and *cis-*9,*trans-*11 18:2 were positively modified in cannulated cows, whose diet was supplemented with a mixture of *Vaccinium vitis idaea* (VVI) and an oil blend (4.83 g VVI/kg and 32.2 g blended oils/kg of DM in total diet, respectively), while 18-carbon FAs in milk of dairy cows were unaffected. Recently, low-molecular-weight compounds, such as anthocyanins and flavanols and CTs from wine lees, were tested, leading to the finding that polyphenols preserve PUFA content during BH, with an important increase in *n-*3 FAs [113]. Once again, there are only limited and confusing data in the literature on the effects of pure molecules on BH. This is the case with the flavonol quercetin, which, at a dose of 500 mg/L, appeared to modulate microbial activity, increasing total VFAs without causing valuable shifts in the pathway or the extent of BH [114]. 

The literature review opened up to a highly fragmented scenario. Attention is particularly focused on the efficacy of tannins on the biohydrogenation process and on the modulation of FA content in the rumen fluid. Plant source, dose, extraction method, and degree of polymerization are factors to be taken into account in the experimental approach. In vitro studies on rumen fluid show positive effects, and the authors often suggest the transfer of the benefit to products for human consumption by ruminants. What is certain is that there is a lack of in vitro protocols that fit the chemical and biological effect, as well as the corroboration of activities through in vivo experiments.

## 5. Effects of Polyphenol Supplementation on Milk Yield and Composition in Dairy Cows

**Figure 8:**
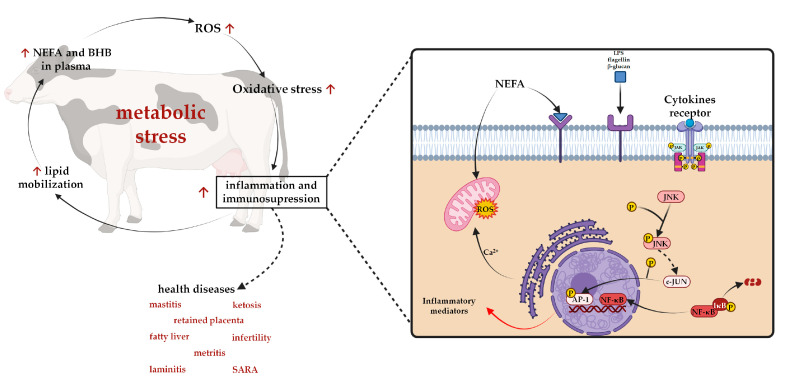
Schematic representation of the main factors affecting the health status of dairy cattle. Different stressors influence the inflammatory responses. Several factors such as non-esterified fatty acids (NEFAs) and β-hydroxybutyrate (BHB) can increase their concentration in plasma, increasing reactive oxygen species (ROS) production and activating pattern recognition receptors (PRRs). In the same way, these receptors could be activated by interactions of endogenous factors such as lipopolysaccharides (LPS), flagellin or β-glucans. Cytokine receptors and certain G-protein receptors also could be activated by different inflammatory mediators. The stimulation of these receptors leads to the activation of two different key transcription factors: activator protein 1 (AP1) and nuclear factor kappa (NF-ΚB) (light-chain-enhancer of activated B cells), which promote the expression of tumour necrosis factor alpha (TNFα), IL-6, IL-1, IL-8, etc. In particular, the phosphorylation of c-Jun leads to translocation in the nucleus and dimerization with *c*-Fos, promoting training of AP-1 transcriptional factor. Instead, the phosphorylation of an inhibitor of kappa B (IκB) leads to its degradation and to the activation of transcriptional factor NF-ΚB [117,118]. Figure created with Biorender.

The physiological change that occurs during the transition period between pregnancy and the peripartum phase is one of the most critical and stressful moments for dairy cows [115]. Overproduction of reactive oxygen species (ROS) leads livestock to oxidative stress, rendering them more susceptible to various diseases (e.g., mastitis, metritis, placenta retention, infertility, SARA; Figure 8) [116]. 

Inflammatory processes occur in dairy cows at the beginning of lactation, when the proteins involved in the process leading to decreased energy are formed and the amino acids necessary for milk production are formed. Therefore, plants rich in polyphenols, supplied as they are or in the form of pure extracts or compounds, capable of exerting an anti-inflammatory effect could ameliorate systemic inflammation by improving milk performance [119,120].

Paper mulberry (*Broussonetia papyrifera*) silage has been shown to have effects on milk production due to its immunomodulatory activity. Indeed, although no improvements in milk yields (MY) have been demonstrated, at dose levels of 10% or 15%, the silage resulted in a significant decrease in the milk somatic cell count (SCC) with improvement in the inflammatory condition [121]. SCC is the most widely used marker of udder health in livestock worldwide and is essential for identifying the occurrence of inflammatory or infectious states in cattle [122]. Its high level is related to oxidative stress and mammary gland infection because the immune system in peripartum dairy cows is suppressed and the animal is more vulnerable. A higher content of free and conjugated total polyphenols has been reported in the blood of animals supplemented with durum wheat bran (3.0 kg/day/cow). The supplementation favoured both the amelioration of oxidative stress status and an improvement in milk quality [123]. Polyphenol detection in plasma also followed long-term grape pomace supplementation (3 months; [124]), whereas dried grape pomace and citrus pulp were proved to provide phenolic compounds also in milk, positively affecting antioxidant performance, and counteracting inflammation in mammary gland [125,126]. The main effects of polyphenols could be achieved in livestock illness reduction, health status and strength improvement, immune system reinforcement and an increase in milk shelf-life [127,128,129,130]. Supplementation with green tea for 45 days highlighted catechin efficiency in restoring health by decreasing oxidative stress biomarkers and proinflammatory cytokines [127], whilst grape pomace (7.5%), supplemented for 60 days, was observed to exert nutrigenomic effects, supplying reinforced immunogenic response [128]. 

The polyphenol dose is the main factor to establish a clear effect on SCC and MY [131], whereas the plant source, independently from a high dose (as in the case Mao pomace meal, *Antidesma thwaitesianum*), could not produce the desired response. Ensiling process reduced polyphenol content, so much so that Cohen-Zinder et al. [129] attributed the improved antioxidant capacity and MY, as well as the low milk SCC, following *Moringa oleifera* supplementation to amino acids and low-molecular-weight peptides. As observed for various other parameters and/or effects, there is no lack of studies aimed at highlighting the efficacy of treatment with various doses and different treatment times with CT and HT on MY. 

Contradictory results describe the ability of polyphenols to increase MY [120,127,131,132,133,134], with the complete presence of significant effects. The need for a long treatment time is referenced in the manuscript of Olagaray et al. [120], who postulated that dietary supplementation with a *Scutellaria baicalensis* extract (10 g/day SBE) improved MY (+ 13%) only if prolonged (60 days). The finding was not confirmed in the most recent report by Grazziotin et al. [131], who suggested that the integration, even at low doses, of tannins and capsicum improved MY (+ 5.3%), or also in the manuscript of Davidovic et al. [132] reporting an MY increase (+ 4.42%) and invariable milk fat and lactose content after supplementing cow diet with tannins (40 g/cow/day). A protective effect of tannins on milk proteins could account for the majority of the effects recorded. 

Tannins from roughage (rice straw and sweet grass) and bamboo grass pellets (*Tiliacora triandra*), as well as those from quebracho-chestnut, increased the protein content and influenced the bacterial population [135,136]. Combining different plants is another strategy pursued, for example, with the combination of extracts of *Quercus robur* L. and *Humulus lupulus* L. benefiting MY (+ 8.11%) and protein levels compared to the sole integration with oak tannins with modifications impacting the fatty acid profile with an increase in PUFAs, mainly LNA, and rumenic acid [134]. In contrast, Herremans et al. [137] found that oak tannin increased MUFA and LCFA (long-chain fatty acids), decreasing saturated fatty acids, SCFA and MCFA (medium-chain fatty acids), with no effect on MY and milk components.

The effects on the variation of milk proteins are probably due to the ability of tannins to form protein–tannin complexes through the formation of weak bonds (hydrogen bonds) stable in rumen pH (5–7) and capable of dissociating at the acid or alkaline pH of the abomasum and duodenum, respectively. The formation of these complexes reduces the degradation of proteins by the rumen microbial population and increases the flow and uptake of amino acids in the small intestine [131]. However, the increased flow is not always associated with the linear increase in milk proteins. Indeed, Estrada-Flores et al. [133] suggested that the use of tannins derived from coffee pulp could affect the production of milk and its proteins by positively improving the flow in the intestine, albeit depending on the dose. They reported that a high dose (1.2 kg/ DM/day of coffee pulp) of tannins can decrease this effect, probably due to the creation of indissociable tannin–protein complexes in the abomasum, making the proteins undegradable. This in turn reduces digestibility, thereby decreasing absorption in the small intestine. Santos et al. [138] also observed that supplementation with Yerba mate (YM; 30 g/kg) and vitamin E (375 IU/kg), despite the source of hydrophilic and lipophilic antioxidants, respectively, did not express a protective effect in milk. Furthermore, supplementation with YM affected the milk protein concentration. Probably, the high levels of tannins in YM could reduce the degradable protein in the rumen, decreasing the supply of amino acids for breast protein synthesis.

Milk yield and its protein content are differently modulated when tannin extracts are supplemented; Acacia mearnsii tannin extract appeared to not affect MY [79,139,140,141,142,143,144] or to negatively impact milk protein [139,140,145,146,147].

Avila et al. [139] showed a decrease in casein (7.11%) with the highest dose of *Acacia mearnsii* CT supplemented, while the antioxidant capacity of milk increased. Analogously, quebracho tannin extract (30 g/kg of DM) reduced MY by 5.8%, also affecting milk protein content [145]. These findings were further confirmed by supplementing a dose equal to 200 g/cow/day of quebracho tannin extract [146], when milk yield was decreased by 14.5%. It was observed that an increase in quebracho-chestnut tannin mixture was related to a linear decline in milk protein content [147], whereas chestnut and quebracho tannic extracts, during different seasonal diets (wet and dry), improved milk quality by enhancing antioxidant and reducing efficacy [148]. Most of the data in the literature reported no significant effects of bayberry and *Acacia mangium* CTs, valonia HTs, chestnut-tannin extract, hazel leaves, and hazelnut skin on MY [149,150,151,152]. However, it has been reported that following the addition of polyphenols, the profile of FAs undergoes significant changes and an influence on the BH process is observed [152]. 

An improvement in the fatty acid profile was also obtained following the integration of propolis with or without vitamin E. Although there were no impacts on MY, protein content, and lactose content, the fatty acid composition was modified with an increase in *trans*-9 18: 1 and *cis*-9, *trans*-11 18:2 instead, and PBP with vitamin E increased *cis*-9, *trans*-11 18:2 and CLA [153]. The most positive finding was the revelation of an increase in total phenols in milk. Although a useful mechanism has not been hypothesized to explain the role of polyphenols in the modulation of the fatty acid profile, the studies conducted to date show that an improvement in the lipid composition of milk, with an increase in polyunsaturated fatty acids, occurs with products enriched in polyphenols [154,155]. Recently, sainfoin polyphenols have been positively correlated with an increase in fatty acids in milk [156,157]. 

There is no lack of conflicting results since previously, *Onobrychis vicifolia* (sainfoin) and *Lotus corniculatus* were investigated without observing changes in milk production, milk fat or protein [156]. However, sainfoin supplementation indicated a modification of fatty acid lipids with an observed increase in FAs belonging to n-3 series [157], increasing protein production in short- and long-term experimental trials, and milk fat, without influencing MY [158]. On the other hand, Moats et al. [159] suggested that tannin use affected milk protein yield, lactose content and yield, while fat content decreased but yield did not. Furthermore, they suggested that the MY in their research was not due to the bean supplement containing tannin because with or without it, the value increased. 

In the light of studies analysed in the last 5 years (Table 1), in some cases the integration of polyphenols, mainly extracts rich in tannins, favours an increase in milk yield of more than 10% [120,133,142,148,158], with a positive impact on the chemical composition of fatty acid, protein and lactose modifications. The positive effect on milk production could also be strongly linked to the ability of these extracts to modulate the stressors that often lead to an inflammatory state during the transition period.

### Milk Enriched in Polyphenols to Improve Human Health and Product Quality

The literature strongly suggests that the composition of milk could be influenced by livestock feeding, enhancing its beneficial effects. Furthermore, diet manipulation could improve performance and milk production during the transition period of dairy cows, as previously described [161].

**Figure 9:**
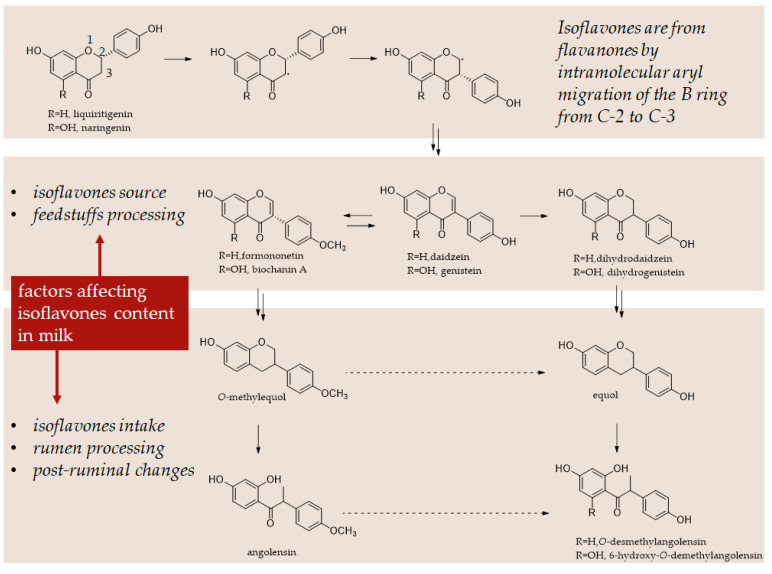
Isoflavone metabolism in plants and animals. The main factors affecting isoflavone content in milk are listed.

However, studies on the bioavailability of polyphenols have been poorly documented. Few authors have reported the presence of several secondary metabolites, mainly isoflavonoids, in milk [162,163,164] suggesting that these beneficial compounds could be absorbed by ruminants with an improvement in the nutritional value of products intended for human consumption (Figure 9). Isoflavonoids are substances with phyto-oestrogenic action, and it has been widely demonstrated that they have an agonist/antagonist effect on oestrogen receptors in both animals and humans. 

Several studies have reported the identification in milk samples of equol, daidzein, and formononentin [165] in different concentrations, strongly dependent on the period and plant-supplemented amount. However, the content of equol in milk is related to the concentration of fat because it and other phenolic compounds can be removed with the milk fat during the skimming process [166].

It is clear from the data available to date that the amounts of these metabolites in milk are influenced by dietary factors, by feeding cows with products containing these compounds, such as flax husks and various legume species. The promotion of beneficial effects is transferred to humans, where an increase in the lignan enterolactone content in the blood is observed [167,168]. Another example is the slightly increased presence of hesperetin and other phenolic compounds in dairy cows fed with citrus pulp [126]. However, the presence of such compounds with oestrogenic activity is partly questionable. It is necessary to further document the possible toxicity of these molecules, especially for more fragile populations such as infants and children in a critical phase of growth, or people who are unable to biotransform some compounds [169].

Furthermore, increasing the levels of polyphenols in milk could also be an approach to improve the shelf life of the product and to prevent the same reactions (e.g., Maillard reactions or Strecker degradation, lipid peroxidation) during the storage process [170].

Another advantage for improving polyphenols in animal products, such as milk and even meat, is their protective activity on n-3 PUFAs. In fact, these are susceptible to peroxidation, and a supply of natural antioxidants, such as polyphenols, should be necessary. Supplementing livestock diets with polyphenols can certainly improve human health, directly or indirectly. However, it is necessary to further evaluate the possibility of transferring these bioactive food molecules into products of animal origin and, above all, to evaluate their bioaccessibility, bioavailability and clearance in order for humans to benefit from them.

## 6. Conclusions

Polyphenols are bioactive compounds, broadly employed as food supplements or nutraceuticals. The use of these compounds for ruminant diet fortification is the focus of the most recent literature. Indeed, their effects are still unclear, as different factors, also comprising their structural complexity and variability, influence their bioavailability. 

It appears clear that polyphenol-induced benefits strongly depend on the animal species and its physiological state, the plant source, the type and dose of polyphenols experimentally applied, as well as their synergistic effects with other coexisting compounds. Indeed, both when investigated as pure compounds or in mixed forms of plant extracts, the definition of the right dose to be administered to achieve beneficial effects is not obvious. Herein, some parameters influenced by the use of polyphenols, through in vitro and in vivo studies, were reviewed. Data in the literature highlight that polyphenols are able to benefit animal health, increasing productive performance. However, the concentrations of potentially active compounds in vitro do not correspond to their bioactivity and/or bioavailability in vivo. In this case, the in vitro approach can be a preliminary, but not absolute, analysis that plays a key role in determining the effects of polyphenol supplementation in the diet of ruminants, such as prevention or mitigation of metabolic diseases, modulation of methanogenesis, with changes in rumen fermentation parameters (e.g., total volatile fatty acids, acetate, butyrate, propionate, N-NH_3_, pH), and guaranteeing quality products. Due to the variety of applications and combinations, metabolomics would broaden our knowledge of how changes in feed nutrition and chemical composition affect rumen metabolism and food quality and composition for humans. Furthermore, the application of different statistical analyses offers the possibility of combining results on rumen fluid, blood or milk by evaluating a possible metabolic correlation. 

It is important to point out that any biological activity exhibited by an extract or a pure molecule is intrinsic to its chemistry. This appears to be the real limitation of the current literature. In fact, analyzing the literature data of this research field, it is evident that several studies investigated the effects of secondary bioactive compounds without actually and extensively analyzing the chemical profile of the supplement in question. Therefore, further investigations focused on the qualitative and quantitative chemical profiles of the matrices and their extracts, considered promising candidates for the integration of livestock, are needed to better clarify the possible effects of polyphenols.

## Figures and Tables

**Table 1:** Milk yield variation (obtained as decreased/increased percentage between milk yield of control group and treated group) and milk composition in terms of fat, protein, and lactose content (%) from different studies. The major increase in milk yield was reported by Estrada-flores et al. [133] through supplementation with coffee pulp (0.6% kg/DM) for 26 days, while the major decrease [146] was observed through supplementation with quebracho tannin extract (200 g/cow/day) for 10 consecutive weeks. ● > −20; ● > −10; ● > −5; −1 < ● < 0; 0 < ● < 1; ● < 5; ● < 10; ● < 20.

		Milk Composition			
Plant/Extract	%Milk Yield vs. CD	Fat %	Protein %	Lactose %	References
*Acacia mearnsii* TE		-	-	-	[88]
		-	-	-	
		-	-	-	
*Acacia mearnsii* TE		2.56	3.03	4.46	[143]
*Acacia mearnsii* TE		2.94	3.37	-	[142]
*Acacia mearnsii* TE		3.59	3.35	-	[141]
*Acacia mearnsii* TE		-	-	-	[139]
		-	-	-	
		-	-	-	
		-	-	-	
*Acacia mearnsii* TE		3.88	3.35	-	[140]
		3.80	3.22	-	
*Acacia mangium* TE		3.33	3.13	4.80	[149]
*Myrica* sp. TE		3.39	3.11	4.81	[149]
*Castanea sativa* TE		3.67	3.32	4.88	[150]
		3.57	3.24	4.77	
*Schinopsis* spp./*C. sativa* TE		3.6	2.92	4.86	[147]
		3.51	2.86	4.87	
		3.57	2.83	4.90	
*Schinopsis* spp./*C. sativa* TE		1.89	1.50	2.27	[136]
		1.85	1.41	2.24	
*Schinopsis* spp./*C. sativa* TE		3.97	3.92	4.6	[148]
*Schinopsis* spp. TE		3.33	3.29	4.69	[145]
		3.43	3.29	4.67	
*Schinopsis* spp. TE		5.93	4.30	5.23	[146]
		6.08	3.94	4.87	
*Quercus robur* TE		4.01	3.26	-	[134]
*Quercus robur* TE		-	-	-	[137]
*Onobrychis vicifolia* TE		1.47	1.12	1.97	[156]
*Onobrychis vicifolia* TE		4.10	3.20	5.00	[157]
*Quercus aegylops* TE		3.42	3.01	4.74	[149]
Tannins/*Capsicum* spp.		3.96	3.03	4.90	[131]
Tanimil SCC		3.73	3.20	4.63	[132]
*Scutellaria baicalensis* FE (trited based on baicalein)		3.84	3.10	4.89	[120]
		3.08	2.89	4.95	
		3.84	3.12	4.95	
		3.29	2.97	4.97	
Propolis extract		-	-	-	[153]
Propolis extract + Vit. E		-	-	-	
Citrus pulp		-	-	-	[126]
Birdsfoot trefoil		3.93	3.14	4.76	[160]
		3.96	3.10	4.76	
		3.78	3.10	4.77	
Coffee pulp		-	-	-	[133]
		-	-	-	
		-	-	-	
Dried grape pomace		3.83	3.32	4.84	[125]
Durum wheat bran		4.05	3.5	4.98	[123]
		4.01	3.6	4.92	
Hazelnut skin		-	-	-	[152]
Mao pomace meal		4.6	3.1	4.5	[130]
		4.4	3.1	4.6	
		4.5	3.2	4.5	
*Onobrychis vicifolia*		4.33	3.69	4.53	[158]
		4.38	3.88	4.57	
		4.20	3.52	4.69	
		4.29	3.75	4.69	
Paper bulberry silage		4.3	3.7	5.1	[121]
		4.2	3.6	5.0	
		3.9	3.6	5.1	
		4.1	3.6	5.1	
Rice straw/bamboo grass pellet		3.5	3.1	4.7	[135]
Sweet grass/bamboo grass pellet		4.0	3.3	4.6	[135]
Yerba mate		-	-	-	[138]
Yerba mate + Vit. E		-	-	-	

(-) not reported; CD = control diet; DM = dry matter; TE = tannin extract; FE = flavonoid extract; st = short-term treatment; lt = long-term treatment.

## Data Availability

Not applicable.
